# Implementation of an advanced practice role for oxygen prescription by physiotherapists in pulmonary rehabilitation: an explanatory sequential mixed-method quality evaluation

**DOI:** 10.1186/s12913-024-12041-5

**Published:** 2024-12-18

**Authors:** Thomas F. Riegler, Thimo Marcin, Patrick Brun

**Affiliations:** 1 Institute of Physiotherapy, School of Health Sciences, ZHAW Zurich University of Applied Sciences, Winterthur, Switzerland; 2 Berner Reha Zentrum, Center for Rehabilitation & Sports Medicine, Insel Group, University Hospital of Bern, University of Bern, Bern, Switzerland; 3 Department for Pulmonary Medicine, Allergology and Clinical Immunology, Inselspital, Bern University Hospital, University of Bern, Bern, Switzerland

**Keywords:** Advanced Practice, Physiotherapy, Oxygen prescription, Oxygen therapy, Oxygen titration, Pulmonary Rehabilitation, Mixed-method

## Abstract

**Background:**

Physiotherapists play a key role in the administration of supplemental oxygen during physical activity in pulmonary rehabilitation. However, supplemental oxygen requires a medical prescription making processes cumbersome for physiotherapists. This study aimed to implement and evaluate an advanced practice role for physiotherapists (APO_2_) allowing them to prescribe oxygen during physical activity.

**Methods:**

Training and certification process for respiratory physiotherapists employed in an inpatient rehabilitation clinic was implemented. A mixed-method approach for retrospective evaluation was used. Quantitative analysis included routine clinical data from oxygen prescriptions, titrations, and exercise capacity. Additionally, healthcare professionals’ experiences and perceptions of the new APO_2_ role was explored using a survey. Qualitative data included interprofessional interviews, survey comments, and data from the critical incidence reporting system.

**Results:**

In 15% of patients during the evaluation period, certified APO_2_ were involved in oxygen prescription. These patients had more frequent titrations (median 8 [interquartile 6, 10] vs. 5 [4, 8]), prescription adjustments (3 [2, 4] vs. 1 [1, 2]), and narrower oxygen dosage ranges prescribed (2 [1, 3] vs. 4 [3, 4]). No significant difference in exercise capacity was observed and no adverse events reported. Survey data from 19 healthcare professionals and interviews indicated that the specialised expertise of APO_2_ positively impacts interprofessional collaboration and workflow efficiency.

**Conclusions:**

Physiotherapy-led oxygen prescription during physical activity in pulmonary rehabilitation is feasible, safe, and perceived as beneficial for the workflow and interprofessional collaboration across healthcare professions.

**Trial registration:**

According to Swiss law (Human Research Act, Art. 2), ethics approval for the study and informed consent were not required and were waived off. All methods were in accordance with the regulations and guidelines of the Swiss Human Research Act and Swiss ethics law.

**Supplementary Information:**

The online version contains supplementary material available at 10.1186/s12913-024-12041-5.

## Background

Physiotherapists play a crucial role in pulmonary rehabilitation by providing individuals with respiratory diseases a range of interventions, including exercise training, breathing exercises, inspiratory muscle training, airway clearance techniques, and lifestyle modifications [1–3]. Hypoxaemia, a prevalent manifestation of lung disease, frequently hampers pulmonary rehabilitation efforts. Additionally, exercise-induced desaturation is often observed in as many as two-thirds of patients during activity [4, 5]. While the administration of oxygen to enhance exercise capacity and alleviate symptoms, particularly in cases of exercise-induced desaturation, remains a topic of debate in patients with COPD [2], its benefits have been demonstrated in individuals with lung fibrosis [6, 7]. Moreover, in patients with pulmonary hypertension, it is common practice to monitor and adjust oxygen therapy during exercise to prevent potential adverse effects, such as syncope [8].

In order to create optimal respiratory conditions for patients during training, it is important to consider factors such as titrated oxygen dosage, suitable oxygen delivery method, possible use of non-invasive ventilation, breathing techniques or airway clearance techniques [9, 10].

Usually supplemental oxygen is classified as medication, and physiotherapists are required to follow medical prescriptions for its indication and dosage [11]. Consequently, oxygen prescription must be authorized, issued, and adjusted by physicians during pulmonary rehabilitation, even if the indication and titration (i.e., individualising required oxygen dosage) are determined by physiotherapists. More specifically, physiotherapists who identify a patient’s need for oxygen supplementation during exercise must obtain a prescription from the treating physician in the standard care setting. The physician then prescribes a certain range of oxygen dosage, within which the therapist performs the titration to find the optimal dosage. If less or more oxygen is needed, a new medical prescription is required from the physician. Furthermore, this complex and time-consuming approach has led to confusion among patients and other healthcare professionals, including nurses, as to whether and how much oxygen is genuinely needed.

To simplify these processes and to reduce the administrative burden for both, physicians, and physiotherapists, this study aimed to implement and evaluate an advanced practitioner role for physiotherapists to empower them to issue institution-specific oxygen prescriptions during physical activity.

## Methods

### Context

A quality assurance project was conducted at the Berner Reha Zentrum, a specialised inpatient rehabilitation clinic located in Berne, Switzerland. The chief physician of the pulmonary medicine department and the head of therapy of the pulmonary rehabilitation collaborated on the definition of an advanced practice role for respiratory physiotherapists called “Advanced Practitioner Oxygen” (APO_2_). Physiotherapists performing the role of an APO_2_ were authorised to indicate and titrate oxygen during exercise and prescribe supplemental oxygen accordingly and adjust target saturation levels in the patient information system without consulting a physician.

To evaluate comparability to standard care and assess the implementation success a pragmatic approach was adopted using an explanatory sequential mixed-method design.

**Fig. 1:**
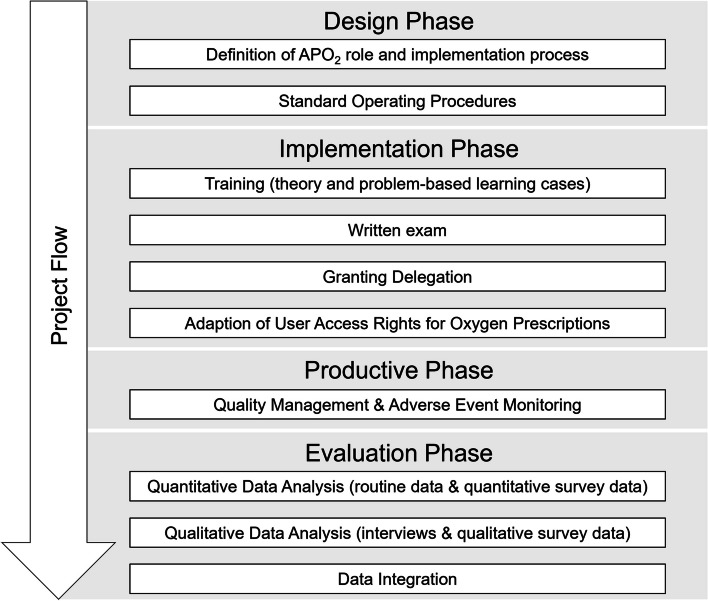
Project flow chart

The productive and evaluation phases lasted from February 2021 to August 2022. The project flow is shown in Fig. 1. This project is a retrospective analysis of anonymised data intended solely for the purpose of improving healthcare processes and maintaining quality standards and does not involve testing of new interventions. According to the Swiss Human Research Act, Article 2, and position papers by swissethics this project is therefore classified as quality assurance [12, 13]. Thus, ethical approval and informed consent was not required and were waived off. All methods were carried out in accordance with the regulations and guidelines of the Swiss Human Research Act and Swiss ethics laws.

### Concept of APO_2_ role

The concept of APO_2_ and its requirements were defined by the chief physician of the pulmonary department and the head of pulmonary therapy on joint agreement. Further, the concept was discussed and approved in interprofessional quality working groups prior to implementation and information was distributed among relevant stakeholders within the institution.

### Requirements

Respiratory physiotherapist with a minimum of two years of experience in treating patients with pulmonary diseases are eligible to undergo advanced in-house training and a final written exam. Upon successful certification therapists are added to the internal APO_2_ registry of the clinic and granted expanded user rights to initiate and adjust the oxygen prescription field in the central prescription document.

### Training and certification

Eligible respiratory physiotherapist underwent a four-month advanced training course given by the head of the pulmonary physiotherapy who has extensive experience in oxygen supplementation. The course was comprised of 12 sessions lasting 2 h each, excluding additional self-study time. The base didactic concept of the course incorporated theory lectures (40% of the course) and problem-based learning cases (60%). The theory lectures focused on the topics oxygen guidelines and their limitations, oxygen systems and delivery methods, advanced blood gas analysis and other laboratory parameters, advanced lung function interpretation, and non-invasive ventilation. Problem-based learning was employed to establish connections between these topics, enhance clinical reasoning skills, and improve decision making skills through argumentation [14, 15]. During the training course, advanced cases were presented by the head of pulmonary therapy. However, trainees were also required to contribute their own clinical cases for discussion and analysis.

The final certification consisted of a two-hour written exam, which included 45 multiple-choice and open-ended questions, that were designed by the head of the pulmonary physiotherapy and reviewed by the chief physician. Questions assessed both theoretical knowledge and the ability to make decisions and provide well-reasoned arguments using clinical cases. To pass the exam and receive the APO_2_ certificate, a minimum of 80% correct answers was required. The certificate was a diploma signed by the chief physician of the pulmonary department, stating the successful certification as APO_2_, and officially granting the delegation mandate.

### Quality management

The definition and responsibilities of the APO_2_ role were documented in standard operating procedures within the clinic’s quality management system.

Certified APO_2_ professionals’ access rights required an adjustment in the patient information system to enable the modification of supplemental oxygen prescription.

In the initial phase, the head of pulmonary therapy and the chief physician provided direct support to APO_2_ when new operating procedures were unclear. Regular meetings between APO_2_ and the head of pulmonary therapy were established to facilitate the sharing of experiences, troubleshoot issues, and ensure ongoing support.

Adverse events were continuously monitored using the critical incident reporting system (CIRS) and were also documented and followed up if reported via informal communication channels such as interdisciplinary quality meetings, email, telephone calls, personal communication, and patient feedback forms. According to standard operating procedures of the Berner Reha Zentrum, adverse events are assessed, and their severity categorised by a dedicated interdisciplinary CIRS team focusing on quality management and improvement. For this project, every incident that involved patients cared for by an APO_2_ was monitored additionally by the head of pulmonary therapy. If an incident is categorised as a serious adverse event related to the APO_2_ program (e.g., wrongful prescription of oxygen that led to symptomatic and serious oxygen desaturation), the project would be halted, and its resumption would be reassessed based on the incident and judgement of the chief physician and the head of pulmonary therapy.

### Evaluation and analysis

One year after the first introduction of APO_2_, quantitative data generated during clinical routine were extracted from the patient information system and anonymized, i.e., all patient identifiable information was permanently removed from the dataset before analysis. Oxygen prescriptions and 6-Minute Walking Test (6MWT) data between APO_2_ and standard care were compared, employing descriptive statistics (median/interquartile ranges and n, %) as well as Pearson’s Chi-squared test and Wilcoxon rank sum test. Statistical analyses were performed using the R version 4.0.3, www.r-project.org [16].

Simultaneously to the retrospective quantitative analyses, a Likert-style survey was sent out to assess views on the APO_2_ role and employer attractiveness. While all APO_2_ were included, only a pragmatic sample of involved physiotherapists, physicians and nurses were approached to participate.

Afterwards, semi-structured interviews with APO_2_, physicians, and nurses were conducted to qualitatively assess their experiences with the new advanced practice role and to gain further perspectives on the quantitative data. All APO_2_ were interviewed, while physicians and nurses were selected based on their involvement with the work of APO_2_ during the pilot period. The number of interviews was limited for reasons of feasibility. Face-to-face, one-on-one interviews were conducted by one investigator (TFR) in quiet and undisturbed rooms. The semi-structured interview guide was developed for this project and designed to identify facilitators and barriers to the APO_2_ concept as well as to gain deeper understanding of the quantitative data. The interview guide is provided in the supplementary material. Recordings were made using RODE Wireless Go and Rode Reporter. The rapid analysis method was employed and an deductive-inductive content analysis was performed [17]. A constructivist worldview was applied for qualitative analyses and final data integration of quantitative and qualitative data [18, 19].

## Results

### APO_2_ therapists

Three eligible respiratory physiotherapists were approached to participate in this pilot project. All three consented to participate and were able to finish the course successfully, subsequently passing the written exam.

### Serious adverse events

No serious adverse events or adverse events concerning APO_2_ were reported through the critical incident reporting system or any other communication channel.

### Quantitative data on oxygen titrations, oxygen prescriptions and 6-Minute walking test outcomes

The retrospective analysis of clinically generated data shows, that the three APO_2_ treated about 15% of patients, aligning with the relative team size. Both groups exhibited similar gender distributions (40% female) and displayed comparable median and interquartile ranges for age (APO_2_-group: 68 years [62, 77], standard care-group: 69 [61, 76]).

Oxygen prescriptions during physical activity were modified more frequently in patients where APO_2_ were involved compared to patients that were treated by regular respiratory physiotherapists only. Notably, the dosage of prescribed oxygen was wider in patients where no APO_2_ was involved as physicians often set the minimum dosage of oxygen prescription to zero.

There were no group differences in 6MWT at entry and discharge. However, improvement was statistically significantly greater in patients with APO_2_ involvement. However, a higher proportion of patients that were treated by APO_2_ required supplemental oxygen during the 6MWTs. Differences in oxygen prescriptions, 6MWT distances and oxygen usage are summarized in Table 1.

**Table 1:** Differences in oxygen prescriptions, 6MWT distances and oxygen usage between patients with APO_2_ involved versus standard care

	APO_2_	Standard care	*p*-value
	*n* = 143	*n* = 866	
Number of oxygen titrations per patient	8 (6, 10)	5 (4, 8)	< 0.001
Number of oxygen prescriptions per patient	3 (2, 4)	1 (1, 2)	< 0.001
Minimum of prescribed oxygen dosage, litres/minute	1 (0, 4)	0 (0, 0)	< 0.001
Maximum of prescribed oxygen dosage, litres/minute	4 (2, 6)	4 (4, 5)	0.029
Range of prescribed oxygen dosage, litres/minute	2 (1, 3)	4 (3, 4)	< 0.001
6MWT start of rehabilitation			
Distance, m	225 (122, 300)	240 (150, 330)	0.2
Number of patients that used oxygen, n (%)	74 (62%)	270 (37%)	< 0.001
6MWT end of rehabilitation			
Distance, m	318 (248, 426)	335 (235, 420)	> 0.9
Number of patients that used oxygen, n (%)	76 (66%)	246 (35%)	< 0.001
6MWT change, m	95 (54, 150)	80 (30, 135)	0.034

Data are given as median (interquartile range). 6MWT: 6-Minute Walking Test

Pearson’s Chi-squared test; Wilcoxon rank sum test

Table 2 shows the oxygen titrations and patients’ free walking distances where the most recent prescription was set by a physician versus prescription set by an APO_2_. The free walking distance is the distance a patient can walk until voluntary exhaustion, which has been tested at least once a week during therapy sessions. For titration purpose, the free walking distance has been tested multiple times with different levels of oxygen dosage.

**Table 2:** Oxygen titrations (testing for needed oxygen dosage) and patients’ free walking distance where the most-recent prescription was set by a physician versus prescription set by an APO_2_

	APO_2_	Standard care	*p*-value
	*n* = 535	*n* = 5848	
Oxygen dosage in range of prescription during titration, n (%)	335 (68%)	4492 (86%)	< 0.001
Saturation in target range during titration, n (%)	255 (56%)	2843 (61%)	0.028
Free walking distance, m	410 (122, 900)	270 (100, 600)	< 0.001

Data are given as n (%) or median (interquartile range)

Free walking distance: Distance a patient can walk until voluntary exhaustion

Pearson’s Chi-squared test; Wilcoxon rank sum test

Physiotherapists performing the APO_2_ role administered oxygen dosages more often outside the dosage range of the current medical prescription during titrations compared to non-APO_2_ physiotherapists (APO_2_, dosage in range: 68%, standard care, dosage in range: 86%), which also led to differences in the oxygen saturation in target range during titrations (APO_2_: 56%, standard care: 61%).

### Quantitative survey data on views about APO_2_ role

A total of 19 healthcare professionals participated in the survey, comprising physiotherapists (*n* = 10), APO_2_ (*n* = 3), physicians (*n* = 3), and nurses (*n* = 3). Remarkably, all respondents unanimously agreed that, from their perspectives, APO_2_ demonstrate competence as oxygen experts, contribute positively to interdisciplinary collaboration, streamline work processes, and elevate the overall quality of treatment. Moreover, 18 of 19 think that oxygen prescriptions by APO_2_ adhere to medical standards. See Table 3 for more details on employer attractiveness and other related factors, as viewed by physiotherapists and APO_2_.

**Table 3:** Survey of APO_2_, physiotherapists, physicians, and nurses on employer attractiveness and feelings about performing this advanced practice role

	APO_2_	Physiotherapists	Physicians	Nurses
	*n* = 3	*n* = 10	*n* = 3	*n* = 3
Prescriptions by APO_2_ follow the medical standard	3 (100%)	9 (90%)	3 (100%)	3 (100%)
APO_2_ are competent experts regarding oxygen prescriptions	3 (100%)	10 (100%)	3 (100%)	3 (100%)
APO_2_ have a positive influence on interprofessional teamwork	3 (100%)	10 (100%)	3 (100%)	3 (100%)
Work processes became easier by introducing APO_2_	3 (100%)	10 (100%)	3 (100%)	3 (100%)
APO_2_ increase the quality of patient treatment	3 (100%)	10 (100%)	3 (100%)	3 (100%)
The possibilities of being an APO_2_ increases employer attractiveness	3 (100%)	9 (90%)	-	-
Carrying out the role as APO_2_ is personally desirable	3 (100%)	6 (60%)	-	-
I think I would be able to make adequate prescriptions without the certification process	2 (67%)	7 (70%)	-	-
The certification process prepared me adequately for the advanced practice role	3 (100%)	-	-	-
I feel up to the task of being an APO_2_	3 (100%)	-	-	-
I feel taken seriously by the interdisciplinary team in my role as APO_2_	3 (100%)	-	-	-
I feel valued by the interdisciplinary team in my role as APO_2_	3 (100%)	-	-	-

Answer options: yes, mostly yes, mostly no, no. Data are given as n (%) of yes and mostly yes

### Qualitative data on interdisciplinary experiences with the APO_2_ role

Three pneumologists, three respiratory nurses, and three APO_2_ participated in interviews to share their experiences with the newly implemented advanced practice role. Ten physiotherapists provided qualitative feedback via the survey's comment section.

*Physicians* provided positive feedback regarding the APO_2_ concept *(“this concept proved itself valuable*,* especially in times of physician shortage*,* it’s easing the burden”*), assured that in APO_2_ therapists *“the knowledge is definitely there” and ”new assistant physicians can learn from APO*_*2 *_*therapists.”* Additionally, they noted that *“patients treated by APO*_*2 *_*are perfectly informed about how to deal with their oxygen.”* Further positive factors were enhanced work process efficiency, clear feedback on patient status and effective teamwork. In contrast, physicians stated that *”guidelines are more important for APO*_*2*_, *than for physicians,”* that *“it needs trust and time to implement concepts like this”* and *“the English model is good*,* but there is need for educational centres”* to really implement this on a broad and reliable spectrum. Additionally, it has been noted, that *“assistant physicians must be protected*,* or there will be only administrative tasks left”*.

*Respiratory nurses* had only good experiences with the new APO_2_ role, adding that *“the constant knowledge is a great advantage for the institution*,* because assistant physicians are changing often”* and they feel that *“APO*_*2 *_*have more knowledge about oxygen*,* than normal physiotherapists and their statements are more reliable.”* Moreover, the narrower ranges on oxygen prescriptions makes it clearer for them to know the patients’ oxygen needs and the oxygen device that will be needed at home. Importantly, hypercapnic patients are identified earlier and the flow of information to nurses is faster. They noted that they wished more physiotherapists would be certified and they feel that APO_2_ represents a quality gain.

*APO*_2_ therapists stated that *“working is more fun,”* not only due to the freedom of prescribing oxygen on their own, but also due to increased knowledge. They appreciate, that there is a team of APO_2_ for peer learning and exchange of experiences. Overall, APO_2_ therapists stated, that they increased their treatment quality (*“I’m able to titrate*,* coach and advise more individually - the self-competency and cardiorespiratory performance of my patients improved clearly”*). However, they expressed that they must communicate wisely with physicians (*“when it comes to oxygen supplementation we are on the same level as physicians. This can lead to tension – it is important to communicate wisely”*) and they feel that there should be salary adjustments due to expanded responsibilities (*“I have more responsibility – this should be reflected in my salary”*).

*Physiotherapists* stated that processes got easier even for them, and they enjoy interesting and insightful conversations and exchanges about their patients with APO_2_. Some stated that they are hopeful that they too can be certified in the future.

### Data integration

A data integration was performed, merging quantitative and qualitative results, employing appropriate data points, and organizing the findings into a joint display. The meta inferences, depicted in Table 4, have been abducted through a comprehensive analysis of the presented data and the specific context in which the project was conducted.

**Table 4:** Joint display and meta-inferences of integrated data from linked quantitative and qualitative results

Quantitative data	Qualitative data	Meta-inference
Significant differences in oxygen dosage in range (68% versus 86%) and saturation in range (56% versus 61%) during titration. Significant differences in number of oxygen titrations per patient. Significant differences in range of prescribed oxygen dosage (2 [1, 3] versus 4 [3, 4] in litres/minute).	Nurses: “Prescriptions are tailored to the patients, are clearer and more explicit – smaller range of dosage”, “[…] easier to understand which oxygen device will be needed at home.”, “APO_2_ have more knowledge about oxygen, than normal physiotherapists and their statements are more reliable” Physicians: “feedback of APO_2_ to questions about patients is very clear” APO_2_: “I’m able to titrate, coach and advise more individually”	APO_2_ test oxygen dosages outside of prescription and document this in the patient information system. Accordingly, smaller oxygen dosage ranges are prescribed. Thus, nurses are better informed and the end-of-rehabilitation procedures are more predictable.
Significant difference in patients’ free walking distance during activities before first required rest (410 m versus 270 m).	Physicians: “patients treated by APO_2_ are perfectly informed how to deal with their oxygen” APO_2_: “I’m able to titrate, coach and advise more individually - the self-competency and cardiorespiratory performance of my patients improved clearly”	APO_2_ feel safer to coach and instructed their patients more individually. Therefore, possibly increasing patient motivation and physical activity by using patient education. Additionally, patients know how to deal with their oxygen more effectively.
No significant or clinically meaningful differences in 6MWT distances. APO_2 _*n* = 2 (67%), physiotherapists *n* = 7 (70%): “I think I would be able to make adequate prescriptions without the certification process.”	APO_2_: “appreciate having a team of APO_2_ for peer learning and exchange of experiences”, “I’m able to titrate, coach and advise more individually - the self-competency and cardiorespiratory performance of my patients improved clearly” Non-APO_2_ physiotherapists: “Easier processes and interesting, insightful conversations with APO_2_”	Possible indication of an already high level of knowledge about the topic of oxygen. Moreover, having an additional team of experts further increases quality of care and sparks interest, as well as knowledge and experience exchange.

## Discussion

This mixed-method quality assurance project investigated the concept of having specialised physiotherapists for issuing in-house oxygen prescriptions during physical activity in pulmonary rehabilitation. No adverse events related to the APO_2_ project were reported during the observational period. Improvements in exercise capacity over the course of rehabilitation were comparable between patients treated with and without APO_2_ involvement. Number of oxygen titrations was higher in patients with APO_2_ involvement while the range of the oxygen prescription was narrower. Overall, physicians, nurses and physiotherapists endorsed the APO_2_ role and considered it as a worthwhile addition to the interprofessional team.

### Oxygen titrations and prescriptions

Advanced Practitioners Oxygen performed significantly more titrations in patients using supplemental oxygen. It can be hypothesised that the increased frequency of testing patients’ oxygen needs, as a standard procedure alongside other treatments delivered by certified APO_2_ therapists, is attributed to increased awareness due to their specialisation.

Moreover, the prescriptions issued by APO_2_ therapists have demonstrated a more precise approach. They exhibit a median minimum dosage of 1 L/minute, offering potentially clearer instructions to other healthcare professionals of when oxygen is warranted during treatment. This streamlined approach proves beneficial in avoiding ambiguity and ensuring appropriate oxygen administration based on patient requirements as supported by the statements from physicians, nurses and APO_2_. Notably, guidelines recommend prioritizing target saturations over specific oxygen dosages [9]. Furthermore, there are statistically significant differences in oxygen dosage and saturation in range during titrations. This suggests that APO_2_ therapists exhibit greater autonomy during this process, potentially enhancing the efficiency of oxygen titration by not relying on physicians’ permissions.

### 6MWT and free walking distance

There were some statistically significant but not clinically meaningful differences in 6MWT distance from start to end of rehabilitation between patients receiving care from APO_2_ compared to standard care, indicating that this rehabilitation outcome is not affected by the APO_2_ concept.

Interestingly, a meaningful difference in free walking distance was observed (APO_2_: 410 [122, 900], standard care: 270 [100, 600], Table 2). It can be hypothesised, that this difference may be attributed to a more individualised patient education by APO_2_ concerning physical activity and instructions on oxygen usage. This hypothesis is supported by interview statements from physicians, nurses and APO_2_ (e.g., *“patients treated by APO*_*2 *_*are perfectly informed about how to deal with their oxygen”*,* “I’m able to titrate*,* coach and advise more individually - the self-competency and cardiorespiratory performance of my patients improved clearly”*). The positive effect of patient education on physical activity has been shown in previous studies [20, 21]. However, these assumptions are based on clinical data in a retrospective analysis and should therefore be considered with caution.

Furthermore, APO_2_ managed more patients with a need for supplemental oxygen during exercise at baseline, which may reflect the referral of patients with supplemental oxygen needs specifically to specialised APO_2_.

### Interdisciplinary teamwork and employer attractiveness

The implementation of APO_2_ therapists aimed to bridge the gap between physicians, physiotherapists, nurses, and patients to improve workflows concerning supplemental oxygen in this clinical setting. Interviews conducted with all involved healthcare professions revealed that APO_2_ therapists were well accepted and valued for bringing constant knowledge and easing the administrative burden, especially in times of staff shortages.

Furthermore, as stated in interviews, the clarity of patients’ supplemental oxygen needs for both nurses and physicians increased, which positively affected interprofessional teamwork. The interprofessional team valued the reliable and specialised knowledge of APO_2_ regarding oxygen supplementation and the interprofessional exchange. Likewise, APO_2_ felt accepted and valued from the interprofessional team. Additionally, in the survey 90% of physiotherapists and 100% of APO_2_ agreed, that the concept of APO_2_ increased employer attractiveness.

An international survey reported that a lack of respect from supervisors and physicians was a key barrier for job satisfaction in advanced practice nurses [22]. There were no issues arising from lack of respect from physicians. Nevertheless, APO_2_ stated the necessity of prudent communication in case physicians are not all aware of the new advanced practice competencies, which could potentially lead to conflicts. This is further reflected in statements from physicians, emphasizing the importance of trust when considering the implementation of advanced practice roles. Further concerns were raised about the potential risk of assistant physicians being relegated to solely administrative roles in rehabilitation. In contrast, it was noted that APO_2_ therapists have effectively reduced the administrative workload for physicians. Moreover, the differing views on guideline usage and fidelity between physicians and APO_2_ serve as another indication for the necessity of prudent and open communication between both parties.

However, training and certification of healthcare professionals requires commitment and time investment from institutions. The survey data show that most physiotherapists are confident that they could theoretically prescribe oxygen during physical activity without a certification process. This perception may be caused by the substantial experience in treating patients with supplemental oxygen in the physiotherapy team. Therefore, it is fair to question if a shortened version of the certification process would be indicated in the future.

### Challenges

Two key challenges have been identified for the continued delivery of the APO_2_ service in this clinical setting. Firstly, APO_2_ expressed the need for increased compensation due to their expanded responsibilities and support provided to colleagues and physicians. However, since this advanced practice role is a novel concept during the pilot phase and not yet established as a regular profession, meeting these demands was not feasible. Notably, research on advanced practice nurses has shown that financial compensation may influence job satisfaction [23]. In contrast to countries like the United Kingdom, Switzerland lacks specific salary structures for advanced practice roles in healthcare professions. Consequently, each healthcare institution’s executive board must individually determine the value of employing advanced practice healthcare professionals.

Secondly, similar to many countries, in Switzerland, advanced practice roles are permitted to fulfil their competencies through delegation policy. In this project we acquired delegation from the head of pulmonary medicine to implement the APO_2_ concept. However, the sustainability of this advanced practice role remains uncertain in the event of changes in key stakeholders.

Considering these challenges, there appears to be a need for a national solution that addresses these issues uniformly and effectively across the country’s healthcare system.

### Strengths & limitations

A strength of this quality assurance project is the individually tailored and close to practice methodology in this specific clinical setting. However, it is important to note that this was not a research study and there were no frameworks to guide the design, implementation, and evaluation of the APO_2_ concept. To the best of the authors’ knowledge there exists only the PEPPA framework, which was designed for advanced practice nurses [24]. While it offers some general guidance, it proved to be insufficiently specific for this application.

Furthermore, it is important to highlight that the quantitative data was not obtained in a controlled research setting but rather derived pragmatically from clinical routine data, which may introduce possible data errors. To address this limitation, qualitative data was incorporated to explore possible explanations and gain a deeper understanding of the quantitative findings.

Additionally, this project was conducted during the COVID-19 pandemic, and it is not clear how the context (e.g., workplace processes) or additional demands from increased numbers of respiratory patients were influenced by it. It can be hypothesized that this circumstance further encouraged the need for modern solutions to ensure high-quality care for patients. Conversely, increased stress due to the higher number of patients could potentially lead to decreased workplace happiness and more errors. However, no specific mentions of the COVID-19 pandemic influencing work satisfaction, workplace processes, or facilitators and barriers to implementing the new role of APO_2_ were noted during the interviews. 

Finally, no interviews with patients were conducted, as this was beyond the scope of the project. Instead, it was chosen to seamlessly integrate the project into existing processes. In hindsight, conducting patient interviews could have provided valuable insights that may have explained differences in free walking distances during activities other than the 6MWT. Additionally, the interviews were conducted by the head of the pulmonary physiotherapy, who designed the APO_2_ concept and was the superior to some of the interviewees, leading to a potential bias.

Further projects may explore the potential expansion of APO_2_ competencies, such as prescription of devices for oxygen at home, indication of non-invasive ventilation during rehabilitation or authorising additional diagnostic tests like arterial blood gases. Moreover, we advocate for the involvement of politicians and professional associations in establishing clear guidelines for salary compensations and legal frameworks for healthcare professionals in advanced practice roles. Such initiatives are crucial in providing appropriate recognition and support for the needed advanced practice roles in the healthcare sector.

## Conclusions

The implementation of physiotherapy-led oxygen prescriptions during physical activity in pulmonary rehabilitation proves to be feasible, safe, and comparable to standard care. Moreover, this approach positively impacts interprofessional teamwork, may enhance employer attractiveness, and holds the potential to promote better informed and more physically active patients.

## Supplementary Information

Supplementary Material 1.

## Data Availability

Data can be requested from the corresponding author (Thomas F. Riegler, thomas.riegler@zhaw.ch).

## Abbreviations

APO_2_: Advanced Practitioner Oxygen
