# Supplementary material for: Implementation of an advanced practice role for oxygen prescription by physiotherapists in pulmonary rehabilitation: an explanatory sequential mixed-method quality evaluation
Source: BMC Health Serv Res. 2024 Dec 18;24:1585. doi: 10.1186/s12913-024-12041-5 (PMC11654414; doi:10.1186/s12913-024-12041-5)
Supplement: Supplementary file 1 — Supplementary Material 1. [file 12913_2024_12041_MOESM1_ESM.docx]

**SUPPLEMENTARY MATERIAL**

**Implementation of an advanced practice role for oxygen prescription** **by physiotherapists in pulmonary rehabilitation: an explanatory sequential mixed-method quality evaluation**

Thomas F. Riegler^1^, Thimo Marcin^2^, Patrick Brun^2, 3^

^1^ Zurich University of Applied Sciences, School of Health Sciences, Institute of Physiotherapy, Winterthur, Switzerland

^2^ Berner Reha Zentrum, Rehabilitation & Sports Medicine, Insel Group, University Hospital of Bern, Bern, Switzerland

^3^ Department of Pulmonary Medicine, Inselspital, Bern University Hospital, University of Bern, Bern, Switzerland

**Semi-structured interview guide for APO2 Quality Evaluation**

Setting and material: Quiet and undisturbed room, recording, and interview guide

Systematics: Rough orientation to the questions in the interview guide; active exploration of new topics introduced by the interviewee.

**Questions for APO2:**

1. How has your function as APO2 affected your daily work routine?
2. In your opinion, were there any advantages APO2 for the professions and patients involved in the treatment plan?
3. Were there any disadvantages to the APO2 role? If so, what were they?
4. How would you describe the perception of your APO2 role by other colleagues?
5. How was the acceptance of your competencies/O2 prescription by nurses and physicians, or other professions?
6. Were there situations where you felt unprepared in your function as APO2?
7. Was the certification process adequate as professional preparation as APO2? If yes, why? If not, what else would have been needed?
8. Is there anything about the certification process that you would want to change?
9. In your opinion, what additional competencies should APO2 physiotherapists acquire?
10. Has the expanded role function had an impact on your professional outlook? If so, how?
11. Is there anything else you wanted to say?

**Questions for Physicians and Nurses:**

1. What are your day-to-day experiences with APO2 and the new processes?
2. Has the implementation of APO2 affected your work? If yes, how?
3. How do you assess the competencies/know-how of APO2 regarding oxygen prescription? What have been your experiences?
4. Are there positive aspects of APO2?
5. Are there negative aspects of APO2?
6. Have you noticed any adverse events regarding the APO2 concept?
7. Is there anything else you wanted to say?

**Additional Question for the Chief Physician:**

1. What motivated you to approve the introduction of the APO2 concept?
